# Evaluation of Retention and Oral Health-Related Quality of Life for Completely Edentulous Subjects Wearing Heat-Cured, 3D-Printed, and Injection-Molded Polyamide Complete Dentures: Randomized Crossover Clinical Trial

**DOI:** 10.3390/dj14020095

**Published:** 2026-02-06

**Authors:** Mohamed Ahmed Helal, Ibrahem M. Ali Abd El Rahman, Ehab Atito, Sara Mohamed Bahaa El-Din, Mostafa Fayad

**Affiliations:** 1 Department of Prosthodontics, Faculty of Dental Medicine, Al-Azhar University, Boys Branch, Cairo 11651, Egypt; 2 MOH, El-Mansoura 35511, Egypt; 3 Department of Removable Prosthodontics, Faculty of Oral and Dental Medicine for Girls, Al-Azhar University, Cairo 11765, Egypt; 4 Department of Substitutive Dental Sciences, College of Dentistry, Taibah University, Al-Madinah 41311, Saudi Arabia

**Keywords:** retention, 3D-printed, polyamide, patient satisfaction, oral health-related quality of life, complete denture

## Abstract

**Objective**: This study aims to evaluate the retentive forces and oral health-related quality of life of completely edentulous subjects wearing heat-cured, 3D-printed, and polyamide complete denture (CD) bases at different intervals. **Subjects and Methods**: For this crossover study, 45 CDs were constructed for 15 completely edentulous male subjects, and subjects were randomly allocated to 3 equal groups (n = 5/group, 3 CDs/subject). Each subject was randomized to receive one manufactured CD—either heat-cured, polyamide, or 3D-printed. After 3 months, subjects crossed over to the other set, with 4 weeks’ rest between each CD. The retentive force (primary outcome) was measured for each maxillary CD base at baseline, after the first and third months; however, the oral health-related quality of life (second outcome) was evaluated for each CD after the first and third months using the oral health impact profile in the completely edentulous patient (OHIP-EDENT) questionnaire. **Results**: There were significant differences in retention forces between the polyamide CD and the other two CDs (*p* < 0.05); however, no significant difference was observed between the heat-cured and 3D-printed CDs at different intervals (*p* > 0.05). After 3 months of follow-up, significant differences in oral health-related quality of life were observed between polyamide and both 3D-printed and heat-cured CDs (*p* < 0.05). Additionally, the comparison between heat-cured and 3D-printed CDs revealed no significant variation in the overall OHIP-EDENT scores (*p* > 0.05). **Conclusions**: The retention of polyamide bases was higher than that of heat-cured and 3D-printed CDs. Additionally, oral health-related quality of life with polyamide dentures was superior to that of 3D-printed and heat-cured CDs across all OHIP-EDENT measures, except for social disability. Both 3D-printed and heat-cured CD bases provide retention and patient satisfaction within acceptable clinical measures.

## 1. Introduction

The need for complete dentures (CDs) is expected to rise steadily over the coming decades. So, improvements in the function and fabrication of complete dentures are still being pursued [1].

Polyamides have gained widespread use as denture base resin materials (DBRMs) due to their benefits, including their superior aesthetic qualities and biocompatible properties, especially for individuals with allergic reactions to resin monomers and nickel elements in traditional metal denture bases. Additionally, compared to conventional resins, they have greater elasticity, reduced water absorption, greater resistance to heat and chemical degradation, and excellent flexural strength. However, one of the main drawbacks of the polyamide denture base is its lack of a chemical bond with the artificial teeth [2,3,4,5].

Recently, there has been a significant integration of computer-aided design (CAD) and computer-aided manufacturing (CAM) within clinical dental practice, especially in prosthesis [6]. Denture processing with CAD-CAM technology can be accomplished through the subtractive method, commonly referred to as milling, or through the additive 3D-printing approach [7].

Three-dimensional printing offers several advantages over both milled and heat-cured CDs, including reduced production time and cost required for denture fabrication; less waste of material; less effort, as many dentures can be printed at the same time; and saved data and designs that can be used for denture duplication if the denture is fractured or lost. The technique allows several copies of the denture to be printed without the need for additional sessions and provides printing for complex designs. It also avoids problems with the drill, such as the size of the drill or the drill being dull, which are limitations of the denture milling technique [8].

The impact of the prosthesis on the well-being of patients in relation to oral health is identified as oral health-related quality of life (OHRQoL), which is a multifaceted concept [9]. The oral health impact profile in completely edentulous patients (OHIP-EDENT) was created by Allen and Locker [10]. It contains nineteen questions with seven subsections: handicap, social disability, psychological disability, physical disability, physical pain, psychological discomfort, and functional limitation.

The resin curing method has a greater influence on the retentive value than the material utilized. The differences included in each stage of the process, such as utilizing CAD software V1.6.3 for design and using the printing software for slicing and throughout the printing of the resin, may have an impact on the retentive properties of the 3D-printing technology [11].

The 3D-printed CDs required higher dislodging forces compared to heat-cured CDs. However, 3D-printed and heat-cured CDs showed a non-significant difference over the 3-month follow-up period [12].

Other studies [13] reported that CD satisfaction is affected by several factors, including adaptation, comfort, speech, aesthetics, and the masticatory function. Additionally, they recorded non-significant differences among genders in achieving satisfaction, and increasing the CD adaptation period led to higher satisfaction among edentulous patients.

Limited data are available in the literature [14,15,16,17,18,19,20,21,22,23]. Thus, a question arises: Do recent methods for complete denture fabrication improve denture quality regarding retention and patient satisfaction? The objectives of this study were to evaluate the retentive forces (primary outcome) and oral health-related quality of life (secondary outcome) of completely edentulous subjects wearing heat-cured, 3D-printed, and polyamide CD bases at predetermined intervals.

The null hypotheses of the current study were that changes in the retention forces and oral health-related quality of life between the heat-cured, 3D-printed, and polyamide CD bases at different time points would be insignificant.

## 2. Subjects and Methods

Sample size calculation was done using a power analysis test based on a prior study [22]; the power was set at 0.80 (1 − β) with a significance level of 0.05 (a confidence level of 95% and effect size 1.31). According to the power analysis, 15 subjects were required for this study.

This clinical study was conducted at the outpatient clinic of the prosthodontic department, Faculty of Dental Medicine for Boys branch, Al-Azhar University, Egypt.

For this study, 15 completely edentulous male subjects were selected with the following criteria: age ranging between 45 and 60 years, healthy cooperative subjects, complete edentulism for at least 6 months, healthy firm mucosa, uniform reduction of the alveolar ridge, free of signs and symptoms of temporomandibular disorders (TMDs), and skeletal class I maxillo-mandibular relation.

Exclusion criteria included subjects with skeletal class II and III maxillo-mandibular relationship, pathological changes of residual ridges, severe undercuts, irregular bony exostosis, flabby, flat, and knife edge ridges, dysfunctions of the masticatory system, parafunctional habits like bruxism, habitual eccentric movements. Hypertensive patients were excluded from this study because both the condition itself and its associated treatments could introduce confounding factors that might affect the study outcomes. Certain antihypertensive medications commonly cause xerostomia, or reduced salivary flow. Saliva is essential for oral health, as it supports oral hygiene, contributes to denture retention through adhesion and cohesion, and helps buffer the oral environment by maintaining pH balance. In addition, patients with debilitating systemic diseases, uncontrolled diabetes, or xerostomia, smokers, and patients currently undergoing or with a history of chemotherapy or radiotherapy were excluded from this study, as both these conditions and their associated treatments could introduce confounding factors that might affect the study outcomes.

### 2.1. Ethical Approval

This study has been approved by the Ethical Committee of the Faculty of Dental Medicine, Boys Branch, Al-Azhar University, Cairo, Egypt, and had approval code (875/281). The protocol was given an ID. NCT06520280 after registration on clinicalTrials.gov (21 July 2024), then subject recruitment started. All patients were informed about the study steps of the research and signed a written consent form for approval.

### 2.2. Study Design

For this crossover study, 45 CDs were constructed for 15 completely edentulous male subjects (3 CDs/subject). Subjects were randomly allocated into three equal groups (n = 5/group). Each group followed a different treatment sequence to minimize order and carry-over effects.

The randomization sequence was generated using an online sequence generator (https://www.randomizer.org/) on (25 July 2024). Three sets of randomization sequences were generated with 5 unique numbers in each set, with numbers ranging from 1 to 15. Only subjects were blinded (single blind study).

The three equal groups were named as follows:

Group 1: heat-cured CDs were constructed and delivered to the subjects of this group.

Group 2: polyamide CDs were constructed and delivered to the subjects of this group.

Group 3: 3D-printed CDs were constructed and delivered to the subjects of this group.

The subjects of each group received three different types of CDs, i.e., three sets of CDs (heat-cured, polyamide, and 3D-printed CDs) were constructed and delivered in a random sequence, with a 4-week rest period between each CD and the others.

The outcomes of this study were as follows:

The retention was measured for each denture base at baseline, after the 1st and 3rd months; however, the OHRQoL was evaluated for each CD after 1st and 3rd months using the OHIP-EDENT questionnaire.

### 2.3. Heat-Cured CD Construction

All conventional steps for the construction of heat-cured CDs were carried out using heat-polymerized acrylic resin (Acrostone Medical and Dental Supplies, Cairo, Egypt).

### 2.4. Polyamide CD Construction

Using conventional methods, another set of maxillary and mandibular master casts was produced for constructing the maxillary and mandibular replica casts (Elite model type III dental stone, Zhermack, Polesine, Italy). The upper and lower recording bases with modeling wax occlusion rims were made for taking the jaw relation records. After that, the face-bow (BIOART equipmento Ltd.a, A7-PLUS Face-bow, Brazil) record was taken for mounting the maxillary cast on the articulator (BIOART equipmento Ltd.a, A7-PLUS Articulator, São Carlos, Brazil). Meanwhile, the lower cast was mounted on the articulator using the centric relation record.

The selected teeth were treated with T-shaped diatoric hole surface treatment, as it increased surface area, and the undercut can improve the mechanical retention and bonding of acrylic teeth to the polyamide denture base, then the setting of teeth was done in balanced articulation [24,25].

During the try-in stage, the aesthetics were checked, and the vertical and horizontal occlusal relationship of the waxed dentures was verified. Then, in accordance with manufacturing instructions, the flasking and injection process of the polyamide dentures (ThermoSens, Vertex Dental, Soesterberg, The Netherlands) was completed using a furnace (Thermoject 22, Vertex Dental, Soesterberg, The Netherlands). The pre-injection temperature reached 272 °C within 15 min. The injection with heating process was performed at a temperature of 272 °C for 1 min, and the pressure inside the furnace was 8 bar.

### 2.5. 3D-Printed CD Construction

For the 3D-printed CD construction, new master cast and recording blocks were produced, and the jaw relationships and face bow records were obtained in the usual manner. The upper and lower casts were mounted on the articulator for setting artificial teeth. The extraoral desktop 3D scanner (DS Mizar scanner, EGS SRL firm, Pescara, Italy) was used to scan the waxed dentures following a successful clinical trial insertion. Using scan spray (BiLKiM Ltd., Izmir, Turkey), the trial dentures and the casts were made ready for scanning in three steps (the try-in, the mandibular cast, and the maxillary cast).

Software for digital design (Exocad GmbH, Darmstadt, Germany) was used to import the standard triangulation language (STL) file. For optimal design, intraoral landmarks were used over the mandibular and maxillary models.

The proper orientation of occlusal relation was done by point alignment of the try-in scan and the cast scan to be accurately superimposed in their proper position. The scans were displayed and stored as STL files.

The position of the occlusal plane was obtained using 3 landmarks on the occlusal record. The maxillary and mandibular casts showed the outline of the denture foundation area. The thickness of the denture bases was determined.

The artificial teeth were removed virtually, and customized sockets were created by software; the individual sockets were customized with available space for resin to be used for teeth bonding.

After completing the design, the teeth and denture base were then exported as two distinct STL files; the designed denture bases and teeth were transferred to the 3D printer software version v2.1.29 (Anycubic photon workshop, Shenzhen, China). The printing angle was fixed at 45 degrees and a layer thickness of 100 microns. The auto support function was used to set supports. The denture bases and teeth were manufactured using Next-Dent liquid (NextDent^TM^, Vertex-dental, Soesterberg, The Netherlands) in accordance with stereolithography technology (SLA).

To generate UV light with a wavelength between 350 and 405 nm, the 3D printer (Anycubic Photon Mono 4K, Shenzhen, China) had a full HD projector, supply chamber, and fabrication chamber. Following the manufacturer’s instructions, the resin liquid was shaken for approximately five minutes before printing the denture bases.

Denture bases and teeth were permitted to suspend on the platform for a brief period after finishing the printing process, and then they were removed with a putty knife.

After brushing and agitating, the printed bases and teeth were immersed in isopropyl alcohol (IPA) ≥ 99% twice. The first was for 3 min, and the second was for two minutes in a different clean bath in a washing machine (Creality 3D UW-01 Washing and Curing Machine 2 in 1 UV Curing Rotary Box Bucket, Shenzhen, China). Afterward, the printed dentures’ base support structures were discarded. Then, the printed object was dried with compressed air properly.

The teeth fit was checked, then the socket surface was coated with resin (same color as the printed one). The teeth were firmly pressed properly inside the base socket. The bond between the denture base and teeth started first at the most posterior molars on each side using a portable UV cure light. Then the teeth support structures were taken out.

**Figure 1:**
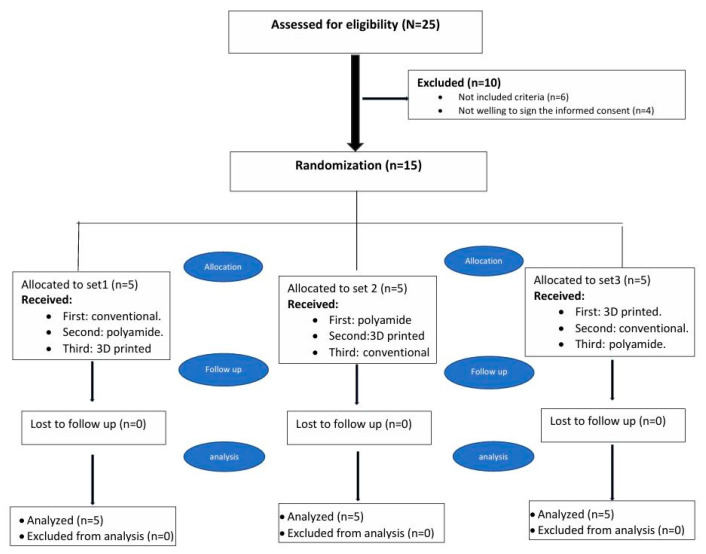
Flow diagram showing the participant enrollment, with the number of participants randomized, allocated to each study set, and dropouts. n—number.

After that, the printed denture was placed in a UV-light curing device (Creality 3D UW-01 Washing and Curing Machine 2 in 1 UV Curing Rotary Box Bucket, Shenzhen, China) after cleaning and drying for 15 min for the final cure. Lastly, a fine bur and rotary tool were used to finalize the 3D-printed denture bases and teeth. The finished prosthesis was then polished using polishing paste (Acrypol, Bredent GmbH, Senden, Germany) and (abraso-Starglanz, Bredent GmbH, Senden, Germany). The last adjustment and recall visits were conducted based on necessity (Figure 1).

### 2.6. Retention (Primary Outcome)

The universal testing machine (Lloyd Instruments Ltd., Bognor Regis, UK) was used to measure CD retention for all patients at the time of delivery, after the 1st and 3rd months after delivery. To ensure that the direction of the dislodgment force was perpendicular to the floor, the patient was informed to sit upright and keep his chin properly positioned over the chin’s support part of the testing device with the occlusal plane parallel to the floor, and the bar of the machine was connected to the upper denture by 4 hooks at equal level attached to the buccal flange with self-cured acrylic resin above the molar and premolar region; both distances between the right and left loop to the geometric center of the denture were similar in values. The exact distance for each loop was checked using an orthodontic wire that was measured using a digital caliper.

The detaching force was increased gradually in the vertical downward direction with a cross-head speed (10 mm/min) until the denture dislodged, and the test was repeated ten times at 5 min intervals (time for denture settling) based on previous studies [26,27], and the mean was calculated. The computer program (Nexygen-MT; Lloyd Instruments, Bognor Regis, UK) recorded the vertical dislodging force, which was recorded by universal testing equipment. The applied force was then stated in Newtons. This test was run on all types of CDs by the same operator.

### 2.7. Oral Health-Related Quality of Life (Secondary Outcome)

The oral health quality of life was evaluated using the primary version of the English OHIP-EDENT (the original 19-item questionnaire) that was developed by Allen and Locker (2002) [10]. The 19 questions and statements of the OHIP-EDENT questionnaire were classified into seven subclasses: functional limitation (D1), physical pain (D2), psychological discomfort (D3), physical disability (D4), psychological disability (D5), social disability (D6), and handicap (D7). A Likert scale was used to answer the questions; the scale ranged from 0 to 4. The scale indicates how frequently an occurrence occurs in a section over a predetermined length of time: never = 0, hardly ever =1, occasionally = 2, fairly often = 3, and very often = 4.

**Figure 2:**
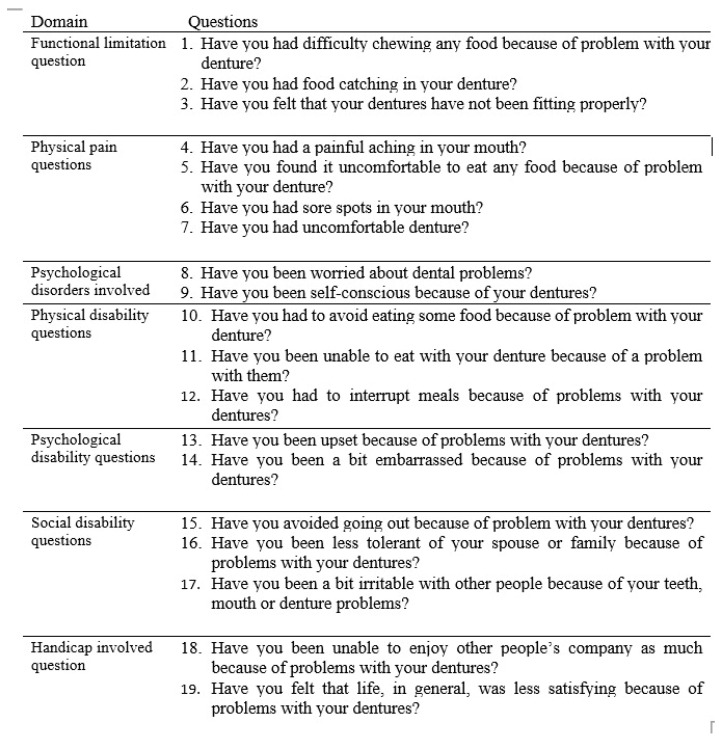
The OHIP-EDENT questionnaire.

Higher scores indicate a stronger adverse impact of oral health issues on quality of life. The score of OHIP-EDENT varies from 0 to 76. Each question response is given a score ranging from 0 (ideal QOL) to 4 (worst QOL). Since the participants were native Egyptian Arabic speakers, the English version of the OHIP-EDENT was translated into Arabic using a forward–backward translation process. The forward–backward translation process was carried out as follows. Two independent certified translators translated the OHIP-EDENT English version into Arabic, firstly using the forward–backward method guidelines stated by Guillemin et al. [28]. Then, the two independent versions were back-translated from Arabic to English. The translated and back-translated versions were compared and discussed by specialists who were excellent in both Arabic and English and had a thorough understanding of OHRQoL assessment. A preliminary Arabic OHIP-EDENT version was then produced. Volunteers then reviewed the primary Arabic versions to identify any issues before the final Arabic OHIP-EDENT version was produced. The questionnaire was given to each patient after delivery for each type of denture base at the 1st and 3rd months (Figure 2).

The Shapiro–Wilk test was used for testing the normality of data. Statistical analysis of the results was performed for parametric data. The retention data were analyzed using a two-way repeated measures ANOVA with Material (polyamide, 3D-printed, heat-cured) and Time (baseline, 1 month, 3 months) as within-subjects factors. Following the omnibus test, simple effects analyses were conducted to examine (1) the effect of time within each material type, and (2) the effect of material at each time point. Pairwise comparisons were performed using Tukey’s HSD test with Bonferroni adjustment. OHIP-EDENT data were analyzed using non-parametric repeated-measures approaches. Intra-group comparisons (changes over time within each material) were analyzed using Friedman’s test with Conover’s post hoc procedure. Inter-group comparisons (differences between materials at each time point) were analyzed using the Aligned Rank Transform (ART) procedure for non-parametric factorial designs with repeated measures. To address multiple testing across 7 domains, results were interpreted with a Bonferroni-adjusted α level of 0.007 (0.05/7). Mean differences with 95% confidence intervals are reported to emphasize clinical effect sizes.

The statistical software SPSS (version 25, IBM Co., New York, NY, USA) was used to analyze the data. *p*-value ≤ 0.05 was considered statistically significant (95% significance level). *p*-value ≤ 0.001 was considered highly statistically significant (99% significance level).

## 3. Results

### 3.1. Retention Forces

The comparative analysis of the retention for the three types of CD bases was carried out at different measuring points (baseline, after 1st and 3rd months of delivery).

Tukey’s post hoc test for pairwise comparisons, comparing between the different measuring points, showed significant variations between the 3-month retention and the other two periods of follow up; however, there were no significant changes between baseline and 1-month follow up. This finding was achieved for polyamide and 3D-printed CDs, but for the heat-cured CDs, there was a significant difference between the three follow up periods, as shown in Table 1.

The overall *p*-value for intra-group comparison was statistically highly significant in the three groups. This significance was related to the difference between the highest mean retention period (after 3 months) and the lowest mean (at baseline).

In the pairwise comparison between the different dentures, there were significant differences between polyamide CD and the other two CDs; however, no significant difference was observed between heat-cured and 3D-printed CDs at the three time intervals.

The overall *p*-value was highly significant for inter-group comparison at all follow-up periods. This is significantly related to the difference between the highest mean retention denture was for polyamide CD, and the lowest mean was for heat-cured CD, as shown in Table 2.

### 3.2. Oral Health-Related Quality of Life

The OHIP-EDENT domains for polyamide, 3D-printed and heat-cured CDs were analyzed, which revealed significant improvements in OHIP-EDENT across all measured parameters between 1- and 3-months follow-up periods.

The analysis for polyamide demonstrated statistically significant improvements across all OHIP-EDENT domains between the two evaluation periods, with varying degrees of significance. While domains of psychological disability, social disability, and handicap showed clear improvements (*p* < 0.05), even more pronounced enhancements were observed in the overall score, functional limitation, physical pain, psychological discomfort, and physical disability of OHIP-EDENT (*p* < 0.001).

Statistical analysis of the 3D-printed CD confirmed improvement, with particularly strong significance (*p* < 0.001) for physical disability, physical pain, psychological discomfort, and the overall OHIP-EDENT score. Social disability, limitation of the function, and handicap domains also showed significant progress (*p* < 0.05), while psychological disability changes did not reach statistical significance.

Statistical analysis of heat-cured CD confirmed improvement, with particularly highly significant (*p* < 0.001) results for functional limitation, physical disability, physical pain, social disability, and the overall OHIP-EDENT score. The psychological disability domain also showed significant progress (*p* < 0.05), while handicap and psychological discomfort changes were statistically nonsignificant, as shown in Table 3.

The comparative analysis of OHIP-EDENT scores was carried out across multiple domains, between the three types of CDs at each follow-up point. One month after delivery, there were significant material-dependent differences in OHIP-EDENT outcomes. The heat-cured CD differed significantly from polyamide in all domains except social disability, while heat-cured CD versus 3D-printed CD comparisons demonstrated significant differences, specifically in functional limitation, psychological disability, handicap, and overall OHIP-EDENT scores.

There were highly significant intergroup differences (*p* < 0.001) for functional limitation, psychological discomfort, physical disability, psychological disability, and overall OHIP-EDENT, along with significant variation in physical pain and handicap domains, but no significant differences (*p* > 0.05) in social disability.

Three months after delivery, statistical analysis identified significant differences between heat-cured and polyamide CDs in all domains except social disability, while comparisons between heat-cured and 3D-printed CDs showed significant variation only in psychological discomfort, handicap, and psychological disability, without significant differences in other domains or the overall OHIP-EDENT score.

These findings demonstrated highly significant intergroup differences (*p* < 0.001) for functional limitation, psychological discomfort, handicap, and overall OHIP-EDENT, along with significant variation (*p* < 0.05) in physical pain, physical disability, and psychological disability. Notably, social disability remained the only domain without significant differences (*p* > 0.05) among the three CD types, as shown in Table 4.

## 4. Discussion

The retention and OHRQoL were reported combined, as denture retention is a significant element that has a significant impact on patient satisfaction and quality of life [29].

Subjects with severe tissue undercuts or irregular ridges were excluded, and the study cast of the patients was confirmed using a digital surveyor to be free from any undercuts, so there was no need to use automatic elimination, which might affect the retention values [12].

Patients with flabby tissues were excluded from the study, as the presence of too much soft tissue allows the denture to slide around the bone, which compromises the stability of the denture bases and lowers their retentive quality [17,30]. Instead of using digital impressions, the current study used conventional impressions, and the master casts were scanned, which was proven to enhance the retention values for entire dentures [31].

Subjects with hypertension were excluded, as anti-hypertensive drugs might cause hyposalivation, and hypertension may be linked to arteriosclerosis and stenosis, which may cause degeneration and hypofunction of the salivary glands and any change in salivary flow might affect denture retention [32,33].

This research used a universal testing machine to measure the retention force to avoid obstacles like anatomical landmarks, which could affect the direction of dislodgment force and prevent it from acting perpendicular to the floor.

The current study results revealed significant differences in the retention and OHRQoL between the different examined CDs, so the null hypotheses were rejected.

The injection molding technique showed the highest retention means compared with traditional processing methods of curing denture bases. This can be explained, as the continuous hydraulic pressure application creates a reservoir for unpolymerized resin that compensates for the shrinkage that occurs during polymerization, which in turn produces superior dimensional accuracy in variance with the conventional compression molding technique [27].

According to the current study, polyamides showed the highest retention values compared with heat-cured and 3D-printed CDs. This increase in retention could be attributed to a variety of variables, including test methodologies, the impact of various processing techniques, and material parameters [16].

The study’s findings demonstrated that the 3D-printed CDs retention forces were greater than those of the heat-cured CDs. These results were consistent with earlier research [12,22,34,35].

The studies by Olawale et al. [14], Afandy [15], Abdullah et al. [22] and Abo Heikal et al. [21] mentioned that retention increased over the follow-up periods. The polyamide, 3D-printed and heat-cured CDs in the current investigation showed an increase in retention value from baseline and after one month and three months. This can be explained by the fact that when the patients’ tissues are allowed to adapt while using and wearing dentures, retention gets better.

According to studies by Abdulkareem & Salem [16] and Afandy [15], polyamide CDs had higher retention forces than heat-cured CDs. These results coincided with the current investigation.

In the current research, the retention values of polyamide and 3D-printed CDs revealed a significant change between 3 months and the other two follow-up periods. It was reported in the study of Abdullah et al. [22] that there were significant changes in the mean retention of the 3D-printed CD with time. Also, Abo Heikal et al. [21] found that there was a statistically significant difference in 3D-printed CDs between baseline and six-month follow-up.

Naggar et al. [17] stated that no significant changes were detected between baseline and after 1 month in retention forces of 3D-printed CDs, which agrees with the current research, which showed a non-significant variation between baseline and 1 month for polyamide and 3D-printed CDs. These may be in accordance with the fact that it may require up to two months for patients to be in harmony with the usage of the denture [36]. However, those findings disagreed with Abdullah et al. [22], who found that there was a significant change in the recorded mean values of retention for the 3D-printed CDs.

A significant difference was recorded between the three follow-up periods of the heat-cured CDs, as recorded by the present study, which was the same finding as studies by Abdullah et al. [22] and Abo Heikal et al. [21].

The investigations of Emera et al. [37] and Behairy et al. [34] concluded that the recorded values of the retention between various periods of follow-up for every group of CD material were statistically significant. These findings were in agreement with our results that showed highly significant retention values in the three denture groups, while it was in contrast with the findings revealed by Hebeshi et al. [12] and Emera et al. [18] who found a non-significant retention increase over the follow-up period within each CD.

The retention of the 3D-printed CD was lower than that of the polyamide CD; however, it was slightly greater than that of the heat-cured CD. This may be due to dimensional changes of the 3D-printed that were caused by residual polymerization and transformation of photo-cured materials, combined with the building up and elimination of supporting structures [38,39]. The precision of the CD base maximizes the retention by optimizing the fit of the CD to the underlying structures, resulting in enhancement of the patient’s masticatory function [40].

A significant effect of the material and method of construction on retention forces of the CDs at different intervals was confirmed by the current research. There was a significant increase in polyamide CDs compared to the other two CDs at different intervals (*p* ≤ 0.05). These were in accordance with previous findings of Kabeel & Kholief [41], Zaky & Abd Elfatah [42] and ELBAMBY et al. [43], who showed that the polyamide CD showed statistically significantly higher means of retention values than heat-cured and digital CDs. Also, Abdulkareem & Salem [16] reported that the retention means of Valplast were significantly greater than conventional denture base materials.

Afandy [15] reported that there was a nonsignificant difference between the retention for nylon and heat-cured CDs, while Olawale et al. [14] found that the retention of heat-cured CDs was higher and statistically significant compared to nylon CDs. These findings were in contrast with the current study results. These might be due to the variance in the retention measuring method between the current study and previous studies, which may affect the retentive force values [44].

In the present study, there were no significant changes in retention forces between heat-cured and 3D-printed CDs at the three time intervals and this agrees with Emera et al. [18], who concluded that there was no statistically significant difference between heat-cured and 3D-printed CDs at each follow-up period. Also, there was disagreement with the studies of Abdullah et al. [22], Hebeshi et al. [12], Abd-Elhaleim et al. [35], Behairy et al. [34], Zohny et al. [23], Naggar et al. [17], and Chebib et al. [19], as their studies reported a significant increase in the retentive value of the 3D-printed CD compared to the traditional CD. These might have resulted from the difference in sample size, technique used for measuring the retention force, neuromuscular control and patients’ adaptability to their dentures.

The OHIP-EDENT was chosen to evaluate the OHQRoL in edentulous patients, as it revealed validity, reliability, and agreement with the complaints reported in many languages. Also, it is considered a useful tool for comparing variables between different countries and cultures [45,46,47].

The CD quality and various methods of CD construction had no effect on patient satisfaction [48].

This study utilized only male subjects, which might be in agreement with the findings of Santos et al. [49], who reported that women scored higher satisfaction than men for aesthetics. This might be a result of the observation that females demand more appointments for denture construction than male patients; additionally, females pay more attention during denture construction, which increases the likelihood of satisfaction following denture delivery.

Patient adaptation to new dentures is extremely variable, as the existence of previous dentures and gender are factors that affect new denture adaptation. Others mentioned that despite the change of a skilled prosthodontist, providing a CD with high quality, the patient was still not fully satisfied with the denture [50].

OHRQoL in edentulous patient rehabilitation with conventional CDs in one or both arches improved after 3 months of use, and maintained its effect for up to 12 months [51].

At the three-month follow-up, in the current study, the polyamide CDs produced the most favorable results across all domains, while heat-cured CDs were correlated with higher OHIP-EDENT scores in all domains except for social disability and physical disability, and overall composite scores. Interestingly, 3D-printed CDs show a higher mean in the domains of social disability and physical disability.

Comparative analysis of the current OHIP-EDENT domains between 1-month and 3-month follow-ups revealed progressive improvements of all three CDs. These results were in agreement with previous studies of Alidema & Sebahate [52] and Keeling et al. [53], who revealed that all the measures of OHIP were enhanced after intervention, regardless of the denture type.

Polyamide demonstrated statistically significant improvements across all OHIP-EDENT domains between the two evaluation periods with varying degrees of significance. These data were in agreement with Alanazi et al. [20], who revealed that the thermoplastic polymers and nylon-based resins enhanced patient comfort and esthetics, and enhanced the functionality of a denture. These might be a result of the flexibility of the polyamide denture material that led to fewer chances of patients complaining of discomfort and irritation, thus improving patient satisfaction.

The 3D-printed CDs showed significance for the overall OHIP-EDENT domain score, except for psychological disability, which revealed a non-significant change. This compromised satisfaction with psychological disability might be related to the features of the printed teeth as monochromatic, while the teeth used with polyamide and heat-cured CDs were multilayered colored teeth [54].

The heat-cured CD revealed significance for all domains, while handicap and psychological discomfort changes did not reach statistical significance. These results might be in accordance with Viola et al. [55], whose findings showed a significant improvement for all OHIP-EDENT domains and heat-cured CDs enriched the OHRQoL for subjects.

The current investigation reported that social disability remained the only domain without significant differences (*p* > 0.05) among the three CD types, suggesting that while material choice substantially impacts most aspects of OHIP-EDENT, its effect on social functioning may be less pronounced by the three-month mark. These results did not match those of Ohara et al. [56], who stated that the OHIP-EDENT of 3D-printed CDs scored significantly higher in social disability.

After 3 months, polyamide CDs showed statistically significant differences with heat-cured and 3D-printed CDs. This agreed with Hazari et al. [57], whose results showed statistical significance in patient satisfaction between nylon CD and the heat-cured CD.

The findings of the current investigation were contrary to those of Ohara et al. [56], who found that 3D-printed and heat-cured CDs had no significant changes in the other domains. However, the 3D-printed CD scored significantly higher than the heat-cured CD in social disability (*p* < 0.05), while in patient satisfaction, the 3D-printed CD was inferior to the heat-cured CD.

There were significant differences after 3 months between heat-cured and polyamide CDs in all domains except social disability, while comparisons between acrylic and 3D-printed CDs showed significant variation only in psychological discomfort, handicap, and psychological disability, with no significant changes in other domains or the overall OHIP-EDENT score. These might be related to the fact that patient satisfaction increased with the improvement of retention and stability of CDs, as it helps dentures to resist under normal functions, enhancing the patient’s confidence, satisfaction and reducing the chances of irritation [58,59].

The final data of the current research concluded a significant variation between 3D-printed CDs and heat-cured CDs after 1 month, which was in agreement with Abdullah et al. [22] and Zohny [23], while those results conflicted with Srinivasan et al. [7], who found no significant changes in OHIP-EDENT readings between the 3D-printed and heat-cured CDs for every single domain, in addition to the overall scores.

After 3 months, the 3D-printed and heat-cured CDs revealed no statistically significant changes, which was in accordance with Hebeshi et al. [60], Iwaki et al. [61] and Abo Heikal et al. [21], who showed no significant changes for patient satisfaction between 3D-printed and heat-cured CDs, which was in contrast to the results of the study by Abdullah et al. [22]. So, it can be stated that using heat-cured and 3D-printed CDs for oral rehabilitation provided improvement in the OHQRoL.

The findings of this research supported the notion that polyamide complete dentures are more retentive and have an excellent impact on the patient’s quality of life, while no difference was found between 3D-printed and heat-cured CDs.

Some limitations of the current study should be considered when interpreting the results. These limitations include the fact that each denture was constructed from a new master cast and new jaw relation records. Additionally, this study was carried out on male subjects. The findings of the current study were limited to 3 months, not long-term evaluation. This time limit was sufficient to assess early clinical performance and patient adaptation to the dentures, but this 3-month follow-up period may not be sufficient to satisfactorily evaluate long-term retention and oral health quality of life because both of the previously mentioned parameters may be affected by the subject’s health status and amount of bone resorption that may be encountered as well. This study suggests a long-term evaluation study with a large number of subjects for the clinical performance of 3D-printed and polyamide CDs.

## 5. Conclusions

Within the limitations of this study, it was concluded that the retention forces of polyamide CDs were higher than those of heat-cured and 3D-printed CDs. Additionally, oral health-related quality of life with polyamide CDs was superior to that with 3D-printed and heat-cured CDs in all aspects of OHIP-EDENT measures except for social disability.

## Figures and Tables

**Table 1:** Value of mean ± SD and intra-group comparison of denture retention results between the different follow-up periods.

CDs	Baseline	1 Month	3 Months	* p * *
**Polyamide**	18.83 ± 2.5 ^b^	20.82 ± 2.44 ^b^	23.69 ± 2.35 ^a^	<0.001 ^HS^
**3D-Printed**	16.31 ± 2.17 ^b^	17.5 ± 1.99 ^b^	20.17 ± 2 ^a^	<0.001 ^HS^
**Heat-Cured**	14.88 ± 0.7 ^c^	16.92 ± 0.2 ^b^	19.1 ± 0.8 ^a^	<0.001 ^HS^

* Overall *p*-value for the simple effect of time within each material group comparison (Simple Effects Analysis following Two-Way Repeated Measures ANOVA). Small letters reflect pairwise comparison between various time intervals (Tukey’s post hoc test), and there is no significant difference between means that share at least one superscript letter at a significance level of *p* ≤ 0.05. HS: highly significant (*p* ≤ 0.001).

**Table 2:** Mean ± SD values, inter-group comparison of denture retention results at different follow-up periods.

CDs	Baseline	1 Month	3 Months
**Polyamide**	18.83 ± 2.5 ^A^	20.82 ± 2.44 ^A^	23.69 ± 2.35 ^A^
**3D-Printed**	16.31 ± 2.17 ^B^	17.5 ± 1.99 ^B^	20.17 ± 2 ^B^
**Heat-Cured**	14.88 ± 0.7 ^B^	16.92 ± 0.2 ^B^	19.1 ± 0.8 ^B^
*p* **	<0.001 ^HS^	<0.001 ^HS^	<0.001 ^HS^

** The ANOVA test’s overall *p*-value for all groups’ intergroup comparison (Simple Effects Analysis following Two-Way Repeated Measures ANOVA). Capital letters for pairwise comparison between different groups (Tukey’s post hoc test), and there is no significant difference between means that share at least one superscript letter at a significance level of *p* ≤ 0.05. HS: highly significant (*p* ≤ 0.001).

**Table 3:** Mean ± SD and intra-group comparison of OHIP-EDENT results between the two time intervals.

CDs	Domains	1 Month	3 Months	Mean Diff [95% CI]	*p* *
**Polyamide**	D1	5.8 ± 1.55	2.5 ± 1.08	3.3 [2.1–4.5]	<0.001 ^HS^
	D2	6.8 ± 2.04	3 ± 1.56	3.8 [2.2–5.4]	<0.001 ^HS^
	D3	2.2 ± 1.14	0.3 ± 0.48	1.9 [1.2–2.6]	<0.001 ^HS^
	D4	3.9 ± 1.6	1.2 ± 0.92	2.7 [1.6–3.8]	<0.001 ^HS^
	D5	2.3 ± 1.25	0.8 ± 0.79	1.5 [0.7–2.3]	0.008 ^S^
	D6	4.5 ± 2.37	1.4 ± 1.26	3.1 [1.5–4.7]	0.006 ^S^
	D7	2.9 ± 1.29	1 ± 0.82	1.9 [1.1–2.7]	0.004 ^S^
	**Overall**	28.4 ± 5.91	10.2 ± 2.94	18.2 [14.3–22.1]	<0.001 ^HS^
**3D-Printed**	D1	7 ± 1.76	4.3 ± 1.42	2.7 [1.5–3.9]	0.004 ^S^
	D2	8.1 ± 1.97	4.5 ± 1.35	3.6 [2.3–4.9]	<0.001 ^HS^
	D3	3.8 ± 1.23	1.9 ± 0.99	1.9 [1.1–2.7]	<0.001 ^HS^
	D4	6.7 ± 0.82	2.8 ± 1.48	3.9 [2.9–4.9]	<0.001 ^HS^
	D5	1.7 ± 0.82	1 ± 0.94	0.7 [−0.1–1.5]	0.096 ^NS^
	D6	4 ± 1.83	1.9 ± 1.29	2.1 [0.7–3.5]	0.016 ^S^
	D7	3.1 ± 1.37	1.5 ± 1.43	1.6 [0.4–2.8]	0.032 ^S^
	**Overall**	34.4 ± 5.85	17.9 ± 5.26	16.5 [11.6–21.4]	<0.001 ^HS^
**Heat-Cured**	D1	9 ± 1.05	5 ± 1.05	4.0 [3.0–5.0]	<0.001 ^HS^
	D2	9 ± 1.05	5 ± 2.11	4.0 [2.4–5.6]	<0.001 ^HS^
	D3	4 ± 1.05	3.5 ± 0.53	0.5 [−0.4–1.4]	0.238 ^NS^
	D4	6.5 ± 0.53	2.5 ± 0.53	4.0 [3.5–4.5]	<0.001 ^HS^
	D5	3.5 ± 0.53	2 ± 1.05	1.5 [0.7–2.3]	0.004 ^S^
	D6	5 ± 1.05	1.5 ± 0.53	3.5 [2.7–4.3]	<0.001 ^HS^
	D7	5 ± 1.05	4 ± 1.05	1.0 [0.0–2.0]	0.064 ^NS^
	**Overall**	42 ± 6.32	23.5 ± 6.85	18.5 [12.3–24.7]	<0.001 ^HS^

* *p*-values from Friedman’s test (non-parametric repeated measures). For interpretation considering multiple testing across 7 domains, results with *p* < 0.007 (0.05/7, Bonferroni-adjusted) should be considered statistically significant. All *p*-values are reported for transparency. Mean differences with 95% confidence intervals emphasize clinical effect sizes. S: *p* ≤ 0.05; HS: *p* ≤ 0.001 (survives domain correction); NS: *p* > 0.05 *.

**Table 4:** Means ± SD and inter-group comparison of OHIP-EDENT results between the three dentures at the different follow-up periods.

CDs	Domains	Polyamide	3D Printed	Heat-Cured	* p * *
**1 Month**	D1	5.8 ± 1.55 ^B^	7 ± 1.76 ^AB^	9 ± 1.05 ^A^	<0.001 ^HS^
	D2	6.8 ± 2.04 ^B^	8.1 ± 1.97 ^AB^	9 ± 1.05 ^A^	0.028 ^S^
	D3	2.2 ± 1.14 ^B^	3.8 ± 1.23 ^A^	4 ± 1.05 ^A^	<0.001 ^HS^
	D4	3.9 ± 1.6 ^B^	6.7 ± 0.82 ^A^	6.5 ± 0.53 ^A^	<0.001 ^HS^
	D5	2.3 ± 1.25 ^B^	1.7 ± 0.82 ^B^	3.5 ± 0.53 ^A^	0.002 ^S^
	D6	4.5 ± 2.37 ^A^	4 ± 1.83 ^A^	5 ± 1.05 ^A^	0.582 ^NS^
	D7	2.9 ± 1.29 ^B^	3.1 ± 1.37 ^B^	5 ± 1.05 ^A^	0.004 ^S^
	**Overall**	28.4 ± 5.91 ^B^	34.4 ± 5.85 ^B^	42 ± 6.32 ^A^	<0.001 ^HS^
**3 Months**	D1	2.5 ± 1.08 ^B^	4.3 ± 1.42 ^A^	5 ± 1.05 ^A^	<0.001 ^HS^
	D2	3 ± 1.56 ^B^	4.5 ± 1.35 ^AB^	5 ± 2.11 ^A^	0.036 ^S^
	D3	0.3 ± 0.48 ^C^	1.9 ± 0.99 ^B^	3.5 ± 0.53 ^A^	<0.001 ^HS^
	D4	1.2 ± 0.92 ^B^	2.8 ± 1.48 ^A^	2.5 ± 0.53 ^A^	0.008 ^S^
	D5	0.8 ± 0.79 ^B^	1 ± 0.94 ^B^	2 ± 1.05 ^A^	0.038 ^S^
	D6	1.4 ± 1.26 ^A^	1.9 ± 1.29 ^A^	1.5 ± 0.53 ^A^	0.748 ^NS^
	D7	1 ± 0.82 ^B^	1.5 ± 1.43 ^B^	4 ± 1.05 ^A^	<0.001 ^HS^
	**Overall**	10.2 ± 2.94 ^B^	17.9 ± 5.26 ^A^	23.5 ± 6.85 ^A^	<0.001 ^HS^

* Overall *p*-value from Aligned Rank Transform ANOVA (non-parametric factorial repeated measures). For interpretation considering multiple testing across 7 domains, results with *p* < 0.007 (0.05/7, Bonferroni-adjusted) should be considered statistically significant. Uppercase letters indicate pairwise comparisons (Bonferroni-adjusted). Means sharing at least one superscript letter are not significantly different (*p* ≤ 0.05). S: *p* ≤ 0.05; HS: *p* ≤ 0.001 (survives domain correction); NS: *p* > 0.05 *.

## Data Availability

The data that support the findings of this study are available on request from the corresponding author. The data are not publicly available due to the privacy of research participants.
